# Awareness of women about cervical smear, human papilloma virus and human papilloma virus vaccine

**DOI:** 10.4274/tjod.galenos.2019.29795

**Published:** 2019-10-10

**Authors:** Emre Başer, Taylan Onat, Demet Aydoğan Kırmızı, Melike Demir Çaltekin, Mustafa Kara, Ethem Serdar Yalvaç1

**Affiliations:** 1 Bozok University Faculty of Medicine, Department of Obstetrics and Gynecology, Yozgat, Turkey

**Keywords:** Human papilloma virus, vaccine, cervical smear, cervical cancer

## Abstract

**Objective:**

The aim of this study was to assess the awareness level of women about cervical smears, human papilloma virus (HPV), and HPV vaccine in a rural city in the central part of Anatolia.

**Materials and Methods:**

A total of 553 patients were included in the study. A 16 item questionnaire developed by our group was completed by all participants. The first part of the questionnaire collected the demographic and socioeconomic information of the participants. In the second part, it was questioned whether this information had a relationship with HPV, HPV vaccine awareness, and cervical screening tests. In the third part, the participants were asked questions related to the acceptance of an HPV vaccine for themselves and their willingness to give consent to have their children vaccinated.

**Results:**

In our study, it was found that HPV awareness significantly increased with the level of education, occupational status and total monthly family income (p<0.001). There was a significant increase in HPV vaccine awareness as the parity (p=0.016), level of education (p=0.025), and occupational status (p=0.001) increased. Having a Pap smear significantly increased with age, income, and number of parity (p<0.001).

**Conclusion:**

Our study revealed that only 9.8% of the women had knowledge about HPV, the majority of the women reported that they would accept vaccination for themselves and for their children. These results indicate that physicians should pay attention to increasing the awareness about HPV.

**PRECIS:** The importance of awareness about cervical smear, HPV and HPV vaccine.

## Introduction

The incidence of cervical cancer is 9 per 100.000 for women in developing countries^(1)^. The rate of cervical cancer has declined dramatically in recent years^(1)^. The most important reason for this is the regular cervical smear screening programs. Although some developed countries have taken these screening programs into routine practice, others have not done so yet; cervical cancer screening rates are much higher in developed countries. For example, the incidence of cervical cancer in the United States was 15 per 100.000 women in 1975, and this incidence was reduced to six per 100.000 women in 2008^(2)^. In Turkey, the rate of cervical cancer has also decreased dramatically after screening programs became widespread. The incidence of cervical cancer was reported as 4.0 per 100.000 in 2014^(3)^. Human papilloma virus (HPV) is the most common sexually transmitted disease agent in the world^(4)^. It causes infections and cancers in many parts of the body. The most common area that is affected by HPV is the cervix. HPV is isolated in 99% of cervical cancers^(5,6)^. Most infections seen in young age are spontaneously cleared by the immune system. Those that are not spontaneously cleansed primarily lead to pre-invasive lesions. Disease that is detected at this stage can be terminated with treatment without conversion to invasive lesions. In addition, HPV vaccination prevents infection with HPV. HPV vaccines have shown an efficacy of 90% in preventing cervical intraepithelial neoplasia 2/3^(7,8)^. The HPV vaccination and smear screening test are highly valuable in the prevention of cervical cancer development. Thus, the most important thing that needs to done is to increase patient awareness. The aim of this study was to assess the awareness level of women about cervical smears, HPV, and HPV vaccines in a rural city in the central part of Anatolia.

## Materials and Methods

This cross-sectional, observational study was conducted at Bozok University Faculty of Medicine, Department of Obstetrics and Gynecology, Turkey. The study protocol was performed according to the principles of the Declaration of Helsinki. After gaining the approval of the Bozok University Ethics Committee, questionnaires were administered through face-to-face interviews to 553 participants in the gynecology clinic. Patients who had a history of HPV infection, had abnormal smear tests or underwent gynecologic surgery were excluded from the study.

A 16 item questionnaire developed by our group was completed by all participants (Table 1). The first part of the questionnaire collected demographic and socioeconomic information of participants, such as age, educational status, educational status of their husbands, total monthly family incomes, occupational status, and number of births (Table 2). In the second part, it was examined whether this information had a relationship with HPV, HPV vaccine awareness, and having cervical screening tests (Tables 3 and 4). In the third part, the participants were asked questions related to the acceptance of HPV vaccination.

### Statistical Analysis

Statistical analysis was performed using the SPSS 20.00 software package (SPSS Inc., Chicago). Descriptive statistics were used to assess patients’ responses. The chi-square test or Fisher’s exact test was applied for categorical variables. P values <0.05 were accepted as significant. The reliability of the questionnaire was assessed by using Cronbach’s alpha coefficients (α). Cronbach’s coefficients range between 0 (weak reliability) and 1 (perfect reliability). We considered 0.7 as the cut-off value indicating acceptable internal consistency for research purposes. An α≥0.8 shows good internal consistency and high reliability.

## Results

The rate of the women aged over 30 years was 51.4%. Educational status was lower in women than men. Although 40% of the women had graduated from primary school, 52% of the men (spouses of the women) had graduated from a middle or high school. When the employment status was investigated, the vast majority of the women were not working (86.6%). The demographic characteristics of the participants are shown in Table 2. HPV awareness was not associated with age and parity (p=0.272 and p=0.299 respectively, and the difference was not statistically significant). However, it was found that HPV awareness significantly increased with levels of education, occupational status, and total monthly family incomes (p<0.050). The awareness of HPV vaccination was significantly higher in women with high parity (p=0.016), but there was no significant difference between the awareness of HPV vaccination and income level (p=0.611). Awareness of HPV and awareness of HPV vaccination were distributed similarly according to occupational status and education level. Both parameters were found to be significantly higher in women with high education level and those with a job (p<0.050). Information associated with HPV and HPV vaccine awareness was shown in Table 3. Two hundred twenty-three participants had a cervical Pap smear at least once before. Having a Pap smear significantly increased with age, income level, and parity (p<0.001, p=0.001, and p<0.001, respectively). However, having a Pap smear was not significantly distributed in relation with educational and occupational status. The previous Pap smear status is shown in detail in Table 4. Only 9.8% of the women had knowledge about HPV. The majority of the women reported that they would accept vaccination for themselves and for their children (Table 5). In addition, HPV awareness was significantly higher in women who had Pap smears (p=0.001). Given that HPV vaccines are not free in Turkey, the women who participated in this study were asked: “If HPV vaccines were free, would you agree to vaccinate yourself, your daughter or your son?” and 56% of the women stated that they would accept the vaccine for themselves, 58% for their daughter, and 59% for their son (Table 5).

## Discussion

Approximately 528.000 women in the world were diagnosed as having cervical cancer in 2012, and about 266.000 patients died of cervical cancer^(9)^. The most important cause of this disease is known to be HPV infection. In the 1970s, the association of HPV with cervical cancer was first detected by Professor Harald zur Hausen. Subsequently, important molecular studies related to HPV have been conducted and its structure described in detail. As a result, prophylactic HPV vaccines have been developed. Prophylactic HPV vaccines were licensed in Europe in the second half of 2006. Quadrivalent and bivalent vaccines have long been marketed in Turkey as well. Although there has been extensive intense debate within the medical community and the media regarding HPV vaccination, it is known that the level of knowledge about HPV in the Turkish community is still very low. Many studies reported that most women were unaware of associated genital lesions such as condyloma and cervical cancer. Unfortunately, no studies have been conducted in recent years to improve awareness of HPV. In a study investigating the knowledge and awareness of the HPV test in the United States, the United Kingdom, and Australia, 50% of all participants reported that they had never heard of HPV^(10)^. In a Serbian study, this rate was reported at about 40%^(11)^. In Turkey, a study conducted in an area of patients with higher socio-economic level found that 45% of women had knowledge about HPV and 40% had knowledge about the causal relationship between HPV and cervical cancer^(12)^. In our study, this rate was found to be much lower (the rate of having information about HPV was 9%). The reason for this difference can be explained by the fact that our study consisted mostly of people living in rural areas. In another study on patients living in rural areas of China, the awareness of HPV was 9.3%^(13)^. This result also supports our opinion. Other than this, this difference is thought to be due to many reasons, such as age, educational status, and religious beliefs.

As age increases, women are less likely to take preventative measures against HPV infection^(14)^. In previous studies among populations of Asian origin, results were found to be similar^(15,16)^. The incidence of cervical cancer screening in general is significantly higher among young women, especially between the ages of 20-35 years^(15,16)^. Although women aged 40 years and over have an increased risk of developing cervical cancer, there is a greater tendency to perceive the cervical examination as taboo in this age group, as well as having more misleading health beliefs and less knowledge about cervical cancer^(17,18)^. In our study, contrary to this information, the rate of having a cervical cancer screening test was found to increase significantly as the age increased. We think the reason for this situation is that as age increases, the fear of gynecologic examination diminishes in relation to the increase in the number of births. As the parity increases, the frequency of referral to the hospital increases. Gynecologists routinely perform cervical screening tests on admission, which increases this rate. We found that the rate of having cervical cancer screening tests in our study was increased with parity (p<0.001), which supports this situation. In a study investigating the relationship between HPV awareness and age, there was no significant difference between women aged under 45 years and women aged over 45 years^(19)^. In our study, no significant relationship was found between age and HPV awareness. This is because the levels of education are at different rates in each age group. According to the World Health Organization’s opinion on HPV vaccination, the recommended primary target population is girls aged 9-14 years, and the secondary target population is older girls or boys. There is no defined age limit for vaccination^(20)^. Due to religious beliefs and social oppression in our country, women may be uncomfortable with talking about sexually transmitted diseases. Therefore, it is important to choose the best words to describe HPV, the HPV vaccine, and cervical cancer screening. The rate of HPV vaccines acceptance is approximately 80-90% worldwide^(21,22,23)^. In studies performed in Turkey, this rate is lower. For example, in a study conducted by Dursun et al.^(12)^ 70% of women stated that they would accept HPV vaccination for themselves, 64% for their daughters, and 59% for their sons. In our study, 56% of the women stated that they would accept the HPV vaccine for themselves, 58% for their daughters, and 59% for their sons. We think that the most important reason why these rates are low is the concerns about religious and social beliefs, and complications of vaccination. In addition, having one sexual partner and practicing safe sex are the main reasons for women’s desire not to have HPV vaccination. Apart from this, an important detail of our study is that no participants had HPV vaccines. We think that the most important reasons for this is the lack of free vaccinations, the lack of adequate information, and the low socio-cultural level of those surveyed. One of the most important factors in health protection is the level of education. For example, in a study conducted among women who have received university education, HPV and HPV vaccine awareness was found to be significantly higher^(12)^. In our study, as the level of education increased, it was found that HPV and HPV vaccine awareness increased significantly. This is supported by a study that found that HPV knowledge was more accessible for women in higher education and metropolitan areas^(13)^. In addition, they reported a higher HPV awareness in women who had previously undergone a Pap smear test in the same study^(13)^. In our study, a similarly high prevalence of HPV awareness was found in women who had previously received a Pap smear. As a result, the importance of the routine application of the Pap smear test arises.

## Conclusion

In conclusion, it is necessary to educate the rural and low-educated population in order to increase the awareness of HPV and the acceptability of the HPV vaccine. In particular, individuals in our society neither take enough care of their own health nor make any attempt to protect their health until disease occurs. However, after disease has occurred, more awareness is created by the information gained in hospitals. Therefore, the most appropriate approach is to make it easier for patients to reach healthcare services.

## Figures and Tables

**Table 1:**
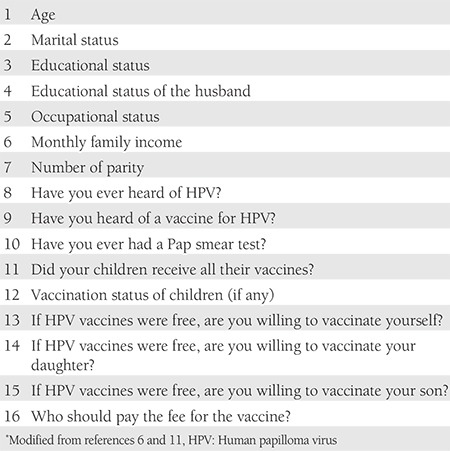
Questionnaire*

**Table 2:**
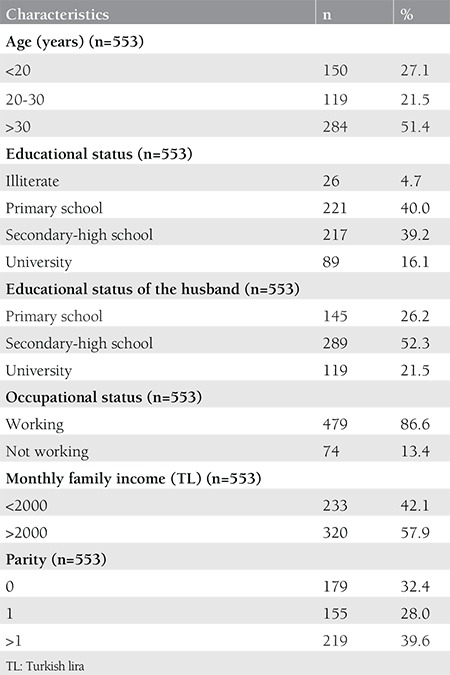
Characteristics of study respondents

**Table 3:**
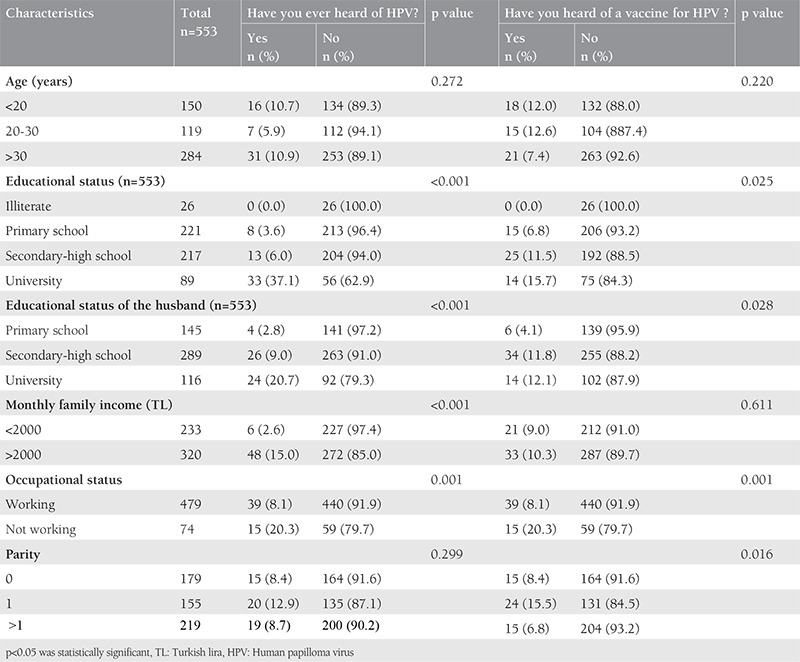
Comparison between the groups regarding knowledge of human papillomavirus and human papillomavirus vaccine

**Table 4:**
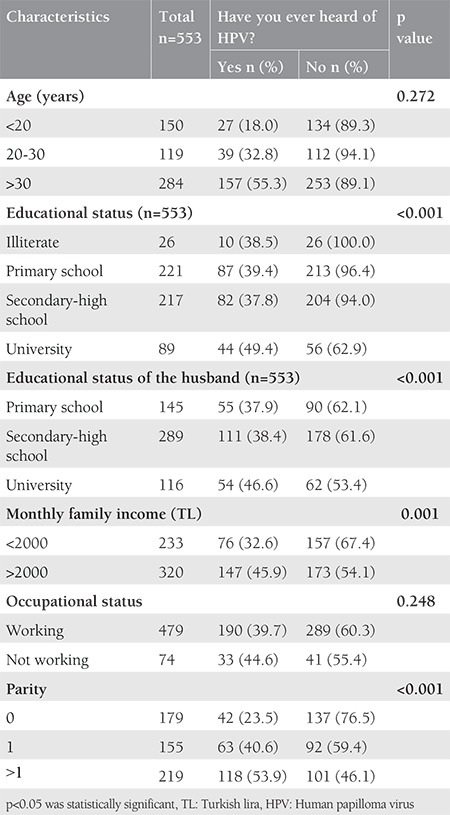
Comparison between the groups regarding status of cervical screening tests

**Table 5:**
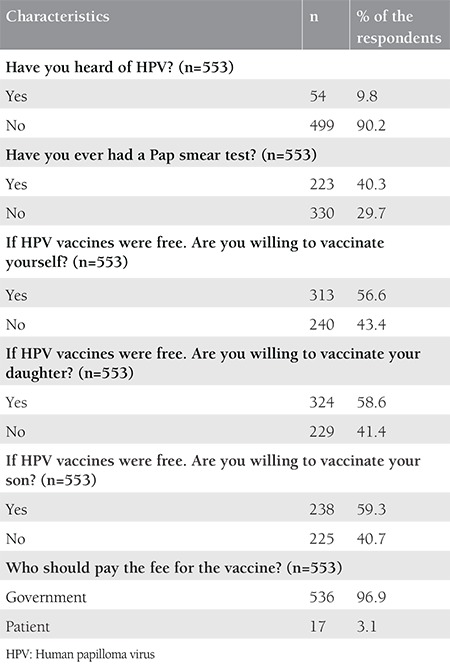
Other results of the survey
